# Screening and identification of miRNAs related to sexual differentiation of strobili in *Ginkgo biloba* by integration analysis of small RNA, RNA, and degradome sequencing

**DOI:** 10.1186/s12870-020-02598-8

**Published:** 2020-08-25

**Authors:** Xiao-Meng Liu, Shui-Yuan Cheng, Jia-Bao Ye, Ze-Xiong Chen, Yong-Ling Liao, Wei-Wei Zhang, Soo-Un Kim, Feng Xu

**Affiliations:** 1 College of Horticulture and Gardening, Yangtze University, Jingzhou, 434025 Hubei China; 2 National R&D for Se-rich Agricultural Products Processing Technology, Wuhan Polytechnic University, Wuhan, 430023 Hubei China; 3 National Selenium Rich Product Quality Supervision and Inspection Center, Enshi, 445000 Hubei China; 4 Research Institute for Special Plants, Chongqing University of Arts and Sciences 402160, ChongQing, China; 5 Department of Agricultural Biotechnology, Seoul National University, Seoul, 08826 Republic of Korea

**Keywords:** Differentially expressed genes, *Ginkgo biloba*, miRNA, Strobili sexual determination, Target gene

## Abstract

**Background:**

*Ginkgo biloba*, a typical dioecious plant, is a traditional medicinal plant widely planted. However, it has a long juvenile period, which severely affected the breeding and cultivation of superior ginkgo varieties.

**Results:**

In order to clarify the complex mechanism of sexual differentiation in *G. biloba* strobili. Here, a total of 3293 miRNAs were identified in buds and strobili of *G. biloba*, including 1085 known miRNAs and 2208 novel miRNAs using the three sequencing approaches of transcriptome, small RNA, and degradome. Comparative transcriptome analysis screened 4346 and 7087 differentially expressed genes (DEGs) in male buds (MB) _vs_ female buds (FB) and microstrobilus (MS) _vs_ ovulate strobilus (OS), respectively. A total of 6032 target genes were predicted for differentially expressed miRNA. The combined analysis of both small RNA and transcriptome datasets identified 51 miRNA-mRNA interaction pairs that may be involved in the process of *G. biloba* strobili sexual differentiation, of which 15 pairs were verified in the analysis of degradome sequencing.

**Conclusions:**

The comprehensive analysis of the small RNA, RNA and degradome sequencing data in this study provided candidate genes and clarified the regulatory mechanism of sexual differentiation of *G. biloba* strobili from multiple perspectives.

## Background

*Ginkgo biloba* L., a perennial gymnosperm, is a living fossil in plant kingdom and called “Gonsun” tree in China. The extract of leaves and nuts from *G. biloba* contains a large number of active compounds, such as flavanol glycosides and terpene lactones, which can be used in the treatment of cardiovascular diseases [1]. At present, *G. biloba* is widely cultivated because it is commonly used in pharmacy, food, skin care products, landscape and other fields [2]. However, the composition of active ingredients in different varieties of *G. biloba* are very divers, which has a significant impact on the quality of *G. biloba* leaf extract. In addition, *G. biloba* is a typical dioecious plant with unique flowering features and has an important role in evolutionary history [3]. It has a long juvenile period and takes almost 30 years to blossom from seedling, which severely affected the breeding and cultivation of superior ginkgo varieties [4, 5]. Therefore, it is particularly important to study reproductive organ development and sexual differentiation of *G. biloba.*

The physiological and molecular mechanism of sexual differentiation and determination of plants have been extensively studied for many years. The mechanism of plant sexual determination mainly includes two systems: genetic sexual determination and environmental sexual determination. Dioecious plants are important materials for studying plant sexual determination and differentiation processes. Up to now, several genes involved in the process of plant sexual determination have been discovered and identified from dioecious species. *Y-gene 1* (*SLY1*) was the first gene associated with sexual differentiation to be cloned and identified from *Silene latifolia* [6]. Subsequently, two MADS-box genes, *SpAPETALA3 and SpPISTILLATA*, were characterized to be involved in formation of male flower primordia in *Spinacia oleracea* [7]. In *Cucumis sativus*, *CsACS2* gene was proved to inhibit male flower formation and promote female flower formation [8]. More recently, two important sex-differentiated genes, *Shy Girl* (*SyGI*) and *Friendly boy* (*FrBy*), were cloned from *Actinidia chinensis* and identified to act for the maintenance of male functions [9, 10]. In addition, development of unisexual male flowers of *Cucumis melo* results from the expression of the sex determination gene, *CmWIP1*, which interacts with *CmbZIP48* to inhibit carpel development [11].

Moreover, the MADS-box transcription factor is closely related to flower organ development and sexual differentiation. For example, five B-type MADS-box homologous genes involved in sexual differentiation were isolated from *Silene alba* but there is no relevant evidence to prove whether these five genes are located on the sexual chromosome [12]. In addition to key genes, most of phytohormones, such as auxin, ethylene (ETH), gibberellin (GA) and cytokinin (CTK), also played important role in sexual differentiation in plant [13]. ETH and CTK mainly promote female flower differentiation and have been widely used in fruit and vegetable industry [14]. IAA (indoleacetic acid) and auxin-like substances mainly act on the elongation of plant cells and promoting female phenotype. Spraying exogenous GA to *C. sativus* increased the male proportion in male and monoecious cucumber and induced the formation of male flowers in gynoecious plants [8]. Therefore, phytohormones play important roles in flower development and sexual differentiation in plant.

microRNA (miRNA) is a kind of endogenous non-coding single-stranded small RNA molecule composed of 18–24 nucleotides that is highly conservative in evolution [15]. Its main function is to regulate the expression level of target genes after transcription by cutting target genes or inhibiting the translation process of target genes [16]. In plants, miRNA participates in the regulation of many life processes, including the regulation of plant growth and development, morphogenesis, organ differentiation, tissue development, signal transduction, flowering and others, as well as the response to biotic and abiotic stresses [17–19]. For example, mutation of DCL1 reduces mature miRNA synthesis that changes the morphology of plant leaves, delays flowering, and leads to female sterility [20]. The growth and development of leaves are affected by miRNA396 in *Arabidopsis thaliana*. Overexpressed miRNA396 resulted in changes in leaf size, decrease of stomatal density and enhancement of drought tolerance [21]. Furthermore, overexpression of miRNA159 led to downregulation of the target genes MYB33 and MYB65, resulting in delayed flowering and male sterility in plants [22, 23]. Also, miRNA plays an important role in plant sexual determination. miRNA172 regulates the sexual differentiation of maize tassel by controlling the expression of the *AP2* gene. In the hermaphroditic maize, miRNA172e targets the IDS1 transcription factor homologous to AP2, thereby preventing the development of pistils in male flowers and the development of intact male flowers. In female flowers, IDS1 transcription factors are not regulated by miRNA172e, so female flowers continue to form a normal pistil [24]. Recently, a Y-chromosome–encoded miRNA has been identified in *Diospyros lotus,* a dioecious plant, can regulate autosome-located *WeGI* gene, responsible for pollen abortion [25].

*G. biloba* is one of representative dioecious trees. Few studies provided information about genetic mechanism of flowering in *G. biloba*. Although our previous work cloned and function reported some genes involved in flower development including three MADS-box transcription factor genes [26–28] and two circadian-regulated genes (*GbCO* and *GbFT*) in photoperiodic pathway [4, 29]. However, little literature reported genetic regulation of sexual differentiation and sexual determination of *G. biloba*, especially for miRNA identification of strobili sexual differentiation and its regulatory mechanism. To further understand the mechanism of strobili sexual differentiation of *G. biloba*, we conducted small RNA sequencing, transcriptome sequencing, and degradation sequencing of male and female buds (MB and FB) as well as microstrobilus (MS) and ovulate strobilus (OS) in this study. Our data identified some important candidate miRNAs and their target genes that regulate sexual differentiation and provided novel insights into genetic mechanism of sexual differentiation of *G. biloba*.

## Results

### Morphological changes of strobili during the flower development

**Fig. 1:**
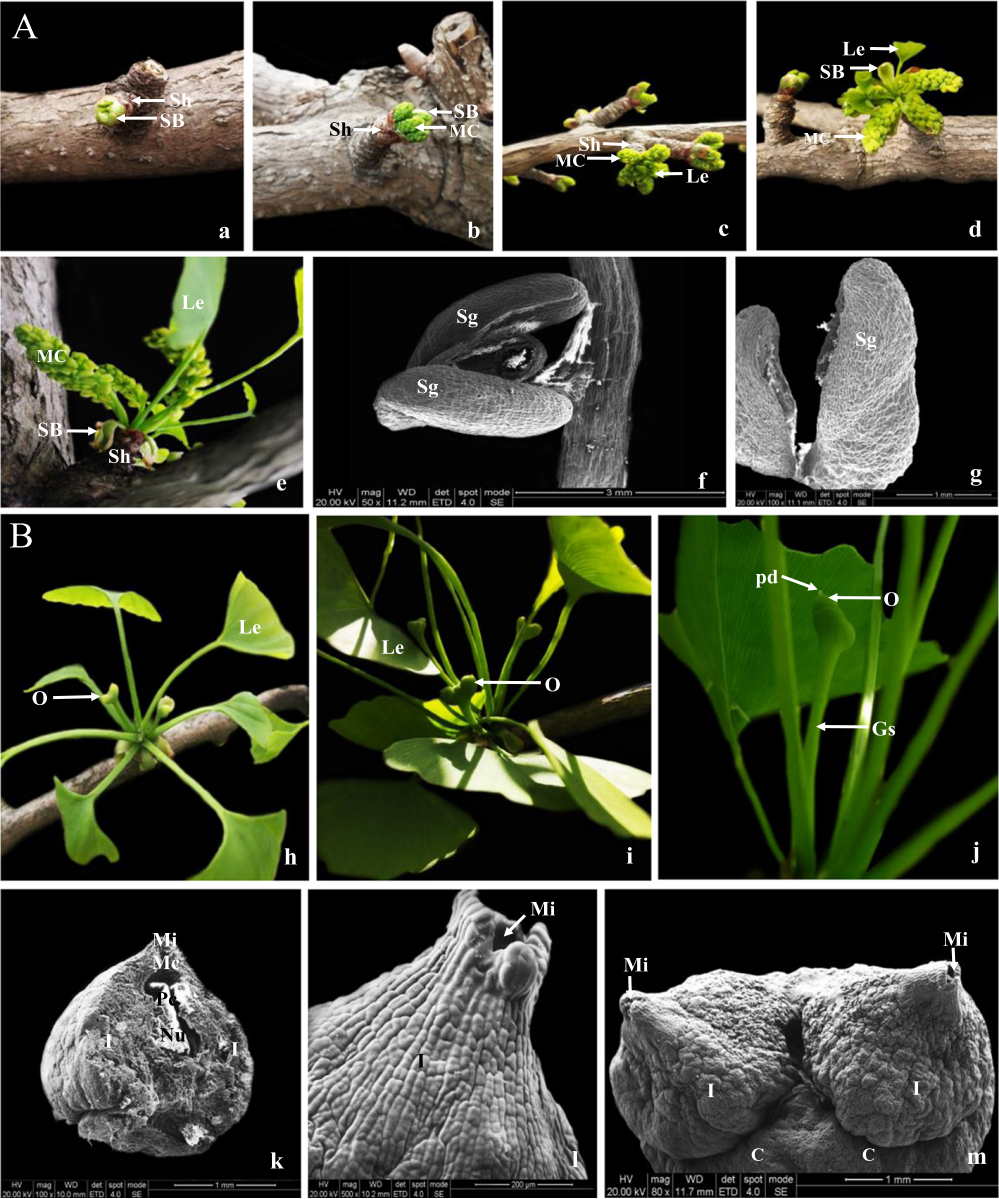
Morphogenesis of female and male cones in *G. biloba*. **a** Morphogenesis of female cones. **a-e**: Male buds gradually unfolded and mature. **f-g**: Male cone. **b** Morphogenesis of male cones. **h-k**: Female flower buds break through the scales until mature ovules. **i-m**: Mature ovules. SB: subtending bract. Sh: short shoot, MC: male cone, Le: foliage leaf, Sg: microsporangium; Le: leaf, O: ovule, Pd: pollination drops, Gs: general stalk, Mi: micropyle, Mc: micropyle canal, Pc: pollen chamber, Nu: nucellus, I: integument, C: body surface

To understand the morphogenesis of female and male strobili of *G. biloba* and their important functions during pollination, the changes in macroscopic and ultrastructure during their development was observed in this study. As shown in Fig. 1, male flower buds are borne at the apex of the short shoots had a dormancy stage of about 6 months. After the dormancy stage, male buds gradually unfolded and mature (Fig. 1a-e). Each male cone contained 60–80 microsporophylls, and each microsporophyll is composed of a sterile extension and two elliptical microsporangia (Fig. 1f-g). Female flower buds are mainly surrounded by thick bud scales until mid-late March, when bud scales began to unfold afterwards. By mid-April, each ovule consisted of a slender stem, an ovule ring, an integument and a nucellus during pollination (Fig. 1h-k). The results of scanning electron microscopy (SEM) showed that ovules differentiated into many structures, including the micropyle, micropylar canal and pollen chamber before pollination (Fig. 1i-m).

### Overview of small RNA deep-sequencing data

For identifying miRNAs involved in the sexual differentiation and development of flower in *G. biloba*, four small RNA libraries of MB, FB, MS and OS were constructed. A total of approximately 79.489 million raw reads were produced. After removing contaminated reads, we obtained clean reads ≥10.78 M from each sample. A total of 3293 miRNAs were obtained from all samples, of which 1085 were known miRNAs and 2208 were novel miRNA, and they were named according to the method of Zhang et al. [30] (Additional file 1: Table S1). All miRNAs sequences were in the range of 18–25 nt, mainly concentrated in 20–24 nt, of which 21 nt was the most abundant type in the small ribonucleic acid library (Additional file 2: Figure S1A). The above-mentioned results are consistent with the typical length of mature miRNA in other plant species [31]. The first base of the 5′ end of the mature miRNA has a strong bias toward U (Additional file 2: Figure S1B). In all base sites, the proportion of bases A and U exceeds 50% except for sites 18 and 19 (Additional file 2: Figure S1C). These statistical results are basically consistent with the characteristics of plant miRNAs, indicating the accuracy of high-throughput sequencing results.

### Differential expression of miRNAs

**Fig. 2:**
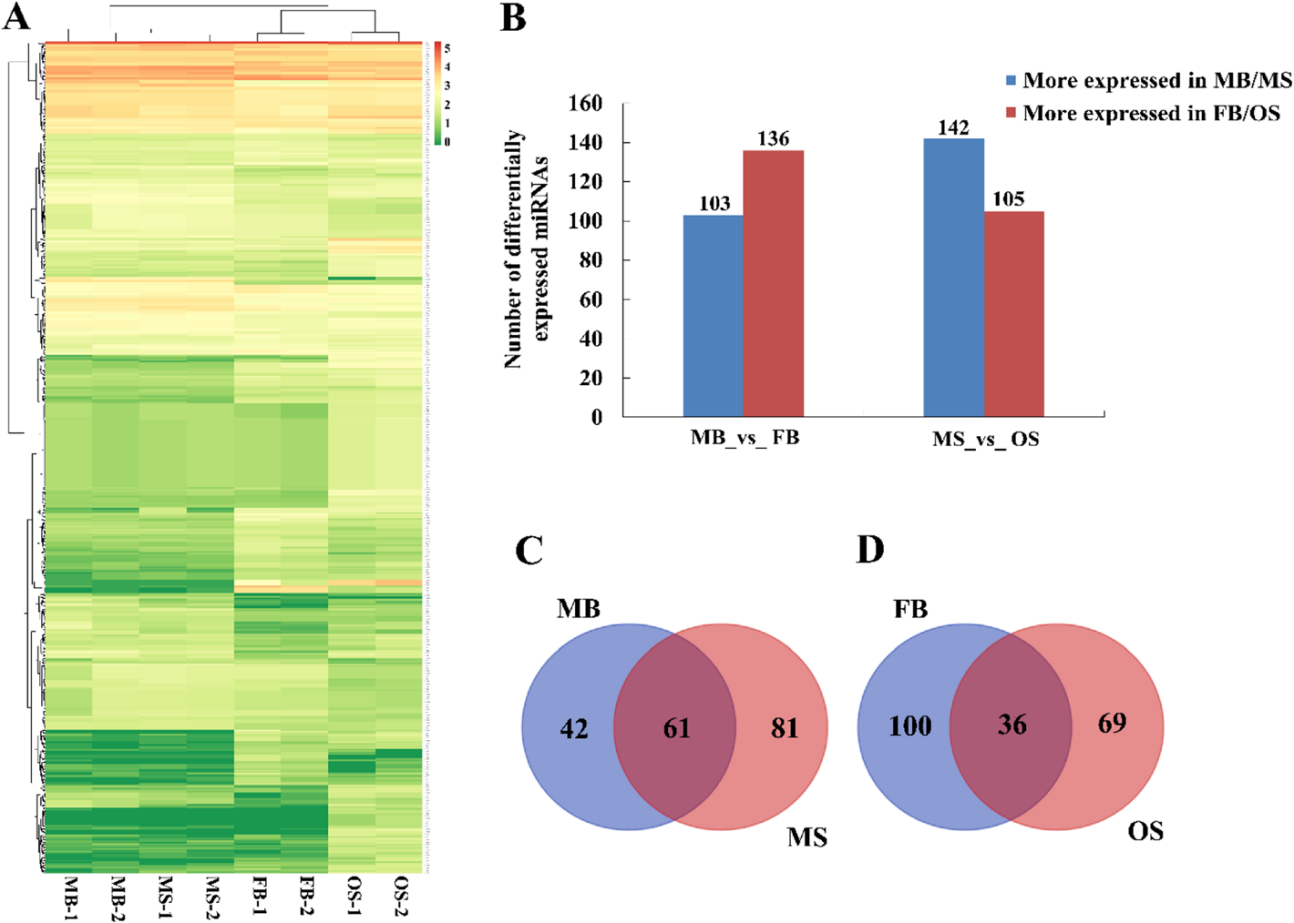
The numbers and expression profiling of differentially expressed miRNAs in strobili of *G. biloba.* **a** Hierarchical cluster analysis of differential expression miRNAs. A scale indicating the color assigned to log2 FPKM is shown to the right of the cluster. Red represents high expression and green represents low expression. Each horizontal bar represents a single miRNA. **b** Statistical histogram of the differentially expressed miRNA in male and female strobili of *G. biloba*. **c** The number of up-regulated miRNAs in male strobili. **d** The number of up-regulated miRNAs in female strobili

False Discovery Rate (FDR) < =0.001and |log2 (Fold Change, FC) | > = 1 were used as the criteria for screening differentially expressed miRNAs. And to study the mechanism of sexual differentiation in *G. biloba* strobili, a hierarchical cluster analysis of the differentially expressed miRNAs was performed, and miRNAs with the same or similar expression behavior were clustered for grouping miRNAs (Fig. 2a). Among 239 differentially expressed miRNAs between MB _vs_ FB, 103 miRNAs more expressed in MB and 136 miRNAs more expressed in FB (Fig. 2b). A total of 247 miRNAs were differentially expressed between MS _vs_ OS, of which 142 miRNAs more expressed in MB and 105 miRNAs more expressed in FB (Additional file 3: Table S2). For analyzing the miRNAs involved in the regulation of sexual differentiation in *G. biloba* strobili, this study mainly analyzed the differentially expressed miRNAs in the same stage. There were 61 miRNAs continuously up-regulated in male strobili and down-regulated in female strobili (Fig. 2c); 36 continuously up-regulated in female strobili and down-regulated in male strobili (Fig. 2d).

### Functional annotation of target genes of differentially expressed miRNA

To investigate the regulatory function of miRNA with respect to target genes, functional annotation was performed on target genes corresponding to differentially expressed miRNA. A total of 3043 target genes out of 6032 genes were annotated to 45 GO categories composed of 13 (2450 target genes, 80.51%), 11 (1308 target genes, 42.98%) and 20 (2129 target genes, 69.96%) categories, which belonged to the molecular function, cellular components, and biological processes, respectively (Additional file 4: Figure S2A). For extending the understanding of the biological functions at the protein level, a total of 2395 target genes were annotated to 25 protein families with classified COG functions (Additional file 4: Figure S2B). Among them, the “general function prediction only” accounted for the largest proportion (627 target genes), followed by “replication, recombination, and repair” (492 target genes). A total of 1251 target genes were successfully annotated to 97 KEGG pathways, and the top 50 metabolic pathways with a higher number of annotated genes were shown in Additional file 4: Figure S2C. In particularly, 60, 59 and 48 target genes were involved in plant hormone signal transduction (ko04075), biosynthesis of amino acids (ko01230) and phenylpropanoid biosynthesis (ko00940), respectively. Enrichment analysis of KEGG pathway of target genes targeted by differentially expressed miRNA showed that Glycophiolipid biosynthesis-globo series (Enrichment factor = 2.48, *q*-value = 0.165) and Starch and sucrose metabolism (Enrichment factor = 1.44, *q*-value = 0.5) pathways were significantly enriched (Additional file 4: Figure S2D). It was worth noting that the enrichment factor of Plant hormone signal transduction pathway was 1.26.

### Screening of Differentially Expressed Genes (DEGs)

**Fig. 3:**
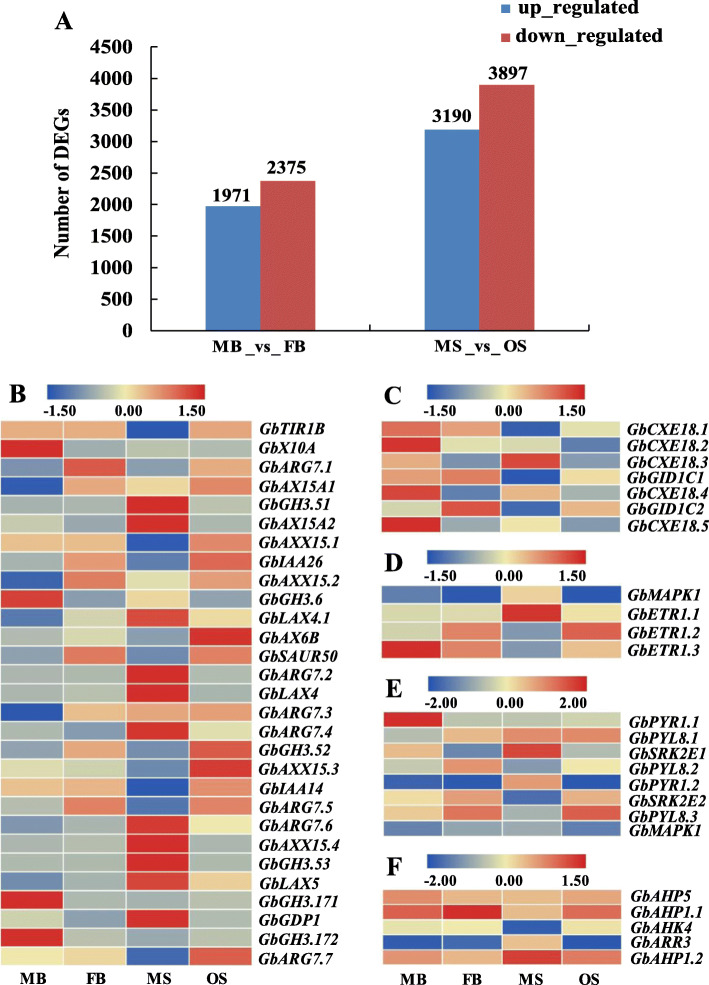
Statistics of DEGs and expression patterns of DEGs in the pathway of hormonal signal transduction. Each column represents different organ of *G. biloba*, whereas each row represents one gene. Expression differences are observed in different colors, and the color scale at the top of each figure represents the log2 FPKM. Red and blue indicate that genes are up-regulated and down-regulated in corresponding organs of *G. biloba*. **a** The numbers of DEGs between organs at different stages. **b** The DEGs in signal transduction pathway of auxin. **c** The DEGs in signal transduction pathway of gibberellin. **d** The DEGs in signal transduction pathway of ethylene. **e** The DEGs in abscisic acid signal transduction pathway. **f** The DEGs in signal transduction pathway of cytokinin

DEGs were obtained and identified by using a Fold Change ≥2 and FDR < 0.01 as screening criteria. A total of 4346 DEGs was screened between MB and FB. Of which, 1971 and 2375 DEGs were up-regulated and down-regulated in FB _vs_ MB, respectively (Fig. 3a). By comparison MS and OS, 7087 DEGs were obtained with 3190 up-regulated and 3897 down-regulated DEGs in OS. These DEGs may play role in phenotype differentiation of male and female strobili in *G. biloba*.

### Regulatory analysis between miRNAs and mRNA

In order to screen the miRNA-mRNA interaction pairs, and the miRNA and mRNA in the interaction pairs were differentially expressed. Using differentially expressed miRNAs as screening criteria, differentially expressed mRNAs regulated by differentially expressed miRNAs were obtained. The results showed that 424 miRNA-mRNA regulatory pairs with up-regulated miRNAs were found in MB, and 493 miRNA down-regulated pairs were observed in MB (Table 1). Among them, 445 pairs had negative correlation (The expression patterns of miRNA and mRNA in the same miRNA-mRNA interaction were opposite in the same organ). A total of 840 miRNA-mRNA regulatory pairs with up-regulated miRNAs were found in OS, and 776 miRNA down-regulated pairs were observed in OS (Table 1). A total of 846 pairs with negative regulatory relationship was screened in MS _vs_ OS (Table 1).

**Table 1:** Number of pairs between differentially expressed miRNAs with their target genes

DEG_Set	Total	Up	Down
MB _vs_ FB	4224	424	493
MS _vs_ OS	5050	840	776

Note: Up, down indicates how the miRNA-regulated target genes were regulated in this group comparison

### Identification of differentially expressed miRNAs and target genes (DEGs) involved in regulating sexual differentiation in *G. biloba*

Many factors play a crucial role in the process of sexual determination. Phytohormones has been proved to be crucial for plant sexual differentiation [32]. Based on the annotation information of target genes and the negative regulatory relationship between miRNAs and mRNAs, we screened candidate miRNA-regulated target genes related to the differential expression of sexual determination in *G. biloba*. As shown in Table 2, five miRNA-mRNA negative correlation pairs were found in the flower development pathway, two pairs of miRNA-mRNA in MB_vs_ FB corresponded to two target genes (*GIGANTEA*, *POX53*), and three pairs of miRNA-mRNA in MS _vs_ OS corresponded to two target genes (*ATR* and *AP2*). In MB_vs_ FB, novel_miR_1881-5p argets the *GIGANTEA and* up-regulated in FB, novel_miR_2912-5p argets the *POX53 and* down-regulated in FB. In MS _vs_ OS, novel_miR_1858-3p targets the *ATR* gene, novel_miR_1944-5p and novel_miR_2329-5p simultaneously target the *AP2* gene, and were down-regulated in OS.

**Table 2:** miRNAs and target genes involved in regulating the process of *G. biloba* strobilus development and sex differentiation

Metabolic pathway /Development	MB _vs_ FB				MS _vs_ OS			
	miRNAID	miR_regulate	mRNATar	Tar_regulate	miRNAID	miR_regulate	mRNATar	Tar_regulate
**Flower development pathway**	novel_miR_1881-5p	up	*GIGANTEA*	down	novel_miR_1858-3p	down	*ATR*	up
	novel_miR_2912-5p	down	*POX53*	up	novel_miR_2329-5p	down	*AP2*	up
					novel_miR_1944-5p	down	*AP2*	up
**GA metabolic pathway**	miR159.1-3p	up	*GAMYB1*	down	miR159.1-3p	up	*GAMYB1*	down
	miR159.1-3p	up	*GAMYB2*	down	miR159.1-3p	up	*SRG1*	down
	miR159.2-3p	up	*GAMYB3*	down	miR159.1-3p	up	*GAMYB4*	down
	miR159.2-3p	up	*GAMYB4*	down	miR159.2-3p	up	*GAMYB4*	down
	miR159.2-3p	up	*GAMYB1*	down	miR159.2-3p	up	*GAMYB1*	down
	miR159.3-3p	up	*GAMYB4*	down	miR159.3-3p	up	*GAMYB4*	down
	miR159.3-3p	up	*GAMYB1*	down	miR159.3-3p	up	*GAMYB1*	down
	miR159.4-3p	up	*GAMYB4*	down	miR159.4-3p	up	*GAMYB4*	down
	miR159.4-3p	up	*GAMYB1*	down	miR159.4-3p	up	*GAMYB1*	down
	miR858-3p	up	*GAMYB1*	down	miR858-3p	up	*GAMYB1*	down
	miR858-3p	up	*GAMYB3*	down	miR858-3p	up	*GAMYB4*	down
	miR858-3p	up	*GAMYB4*	down	novel_miR_2221-3p	up	*GALMADRAFT*	down
	miR319.1-3p	up	*GAMYB3*	down	novel_miR_3455-3p	up	*GALMADRAFT*	down
	miR319.2-3p	up	*GAMYB3*	down	novel_miR_1944-5p	down	*PME13*	up
	miR319.3-3p	up	*GAMYB3*	down	novel_miR_2329-5p	down	*PME13*	up
	novel_miR_1872-3p	up	*ARHGAP7*	down				
**ETH metabolic pathway**	miR2950.1-3p	up	*ERF2*	down				
	miR2950.1-5p	up	*ERF2*	down				
**IAA metabolic pathway**					miR160.1-5p	up	*ARF18.1*	down
					miR160.1-5p	up	*ARF18.2*	down
					miR160.2-5p	up	*ARF18.1*	down
					miR160.2-5p	up	*ARF18.2*	down
					miR160.3-5p	up	*ARF18.1*	down
					miR160.3-5p	up	*ARF18.2*	down
					miR160.4-5p	up	*ARF18.1*	down
					miR160.4-5p	up	*ARF18.2*	down
					miR160.5-5p	up	*ARF18.1*	down
					miR160.5-5p	up	*ARF18.2*	down
					miR160.6-5p	up	*ARF18.1*	down
					miR160.6-5p	up	*ARF18.2*	down
**CTK metabolic pathway**					miR2950.3-5p	up	*AHP5*	down

A total of 23 pairs of negative regulatory relationships were involved in GA metabolic pathway, 16 pairs and 5 target genes (*GAMYB1*, *GAMYB2*, *GAMYB3*, *GAMYB4*, *ARHGAP7*) in the MB _vs_ FB; 15 pairs and 5 target genes (*GAMYB1*, *SRG1*, *GAMYB4*, *GALMADRAFT*, *PME13*) in the MS _vs_ OS. In addition, eight known miRNAs (miR159.1-3p, miR159.2-3p, miR159.3-3p, miR159.4-3p, miR858-3p, miR319.1-3p, miR319.2-3p, and miR319.3-3p) and novel_miR_1872-3p were up-regulated expressed level of observed in the FB; two novel miRNAs (novel_miR_2221-3p and novel_miR_3455-3p) were up-regulated expressed level, and novel_miR_1944-5p and novel_miR_2329-5p were down-regulated in OS (Table 2). The corresponding target genes were transcription factor GAMYB (*GAMYB1, GAMYB2, GAMYB3, GAMYB4*) and other genes involved in plant GA metabolic pathway (*ARHGAP7, GALMADRAFT*), which were down-regulated; however, the *PME13* was up-regulated in OS.

For the ETH metabolic pathway, two miRNA-mRNA pairs were observed to negatively correlate in MB _vs_ FB, corresponding to one target gene (*ERF2*), and down-regulated expression in FB.

In the MS _vs_ OS, twelve pairs annotated to the metabolic pathways of IAA and two target genes (*ARF18.1*, *ARF18.2*). miRNA160.1-5P, miRNA160.2-5P, miRNA160.3-5P, miRNA160.4-5P, miRNA160.5-5P, miRNA160.6-5P were up-regulated in OS. The corresponding target genes were auxin response factor 18 (*ARF18.1*, *ARF18.2*), which were down-regulated.

According to the analysis of transcriptome data, 235 DEGs were annotated to the plant hormone signal transduction pathway, of which 81 DEGs were in the MB _vs_ FB and MS _vs_ OS. The expression patterns of genes annotated to IAA, GA, ETH, CTK and the ABA signal transduction pathway were analyzed to find out that there were 52 DEGs, of which 29 DEGs were in the IAA signal transduction pathway (Fig. 3b), 7 DEGs in the GA signal transduction pathway (Fig. 3c), 4 DEGs in the ETH signal transduction pathway (Fig. 3d), 8 DEGs in the ABA signal transduction pathway (Fig. 3e), and 5 DEGs in the CTK signal transduction pathway (Fig. 3f). These DEGs involved in hormone signal transduction may participate in sexual determination of *G. biloba*. Combined with the negative correlation regulation between miRNA and mRNA, only 1 of the 52 DEGs had a negative correlation regulation, namely, *AHP5* involved in the CTK signal transduction pathway. In OS, the gene was down-regulated while the corresponding miRNA (miR2950.3-5p) was up-regulated (Table 2). This miRNA may play an important regulatory role in flower differentiation and sexual differentiation of *G. biloba*.

### Comparison of phytohormone contents of female and male strobili of *G. biloba*

**Fig. 4:**
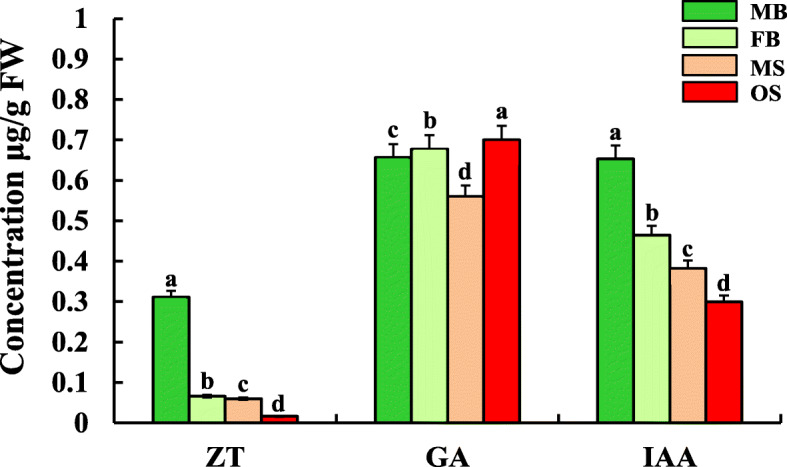
Hormone content in male and female strobilus of *G. biloba*. Data of hormone content were shown as mean ± SE (*n* = 3). The different letters indicates values with significantly different at *P* < 0.05

Zeatin (ZT), GA_3_ and IAA were detected at different developmental stages of the female and male strobili of *G. biloba* (Fig. 4). It was worth mentioned that the change trend of hormone contents was consistent in two developmental stages of male and female strobili. In detail, the contents of ZT and IAA in male strobili were significantly higher than those in female strobili, while the contents of GA_3_ in female strobili were significantly higher than those in male strobili (Fig. 4).

### Confirmation via degradome approaches

**Fig. 5:**
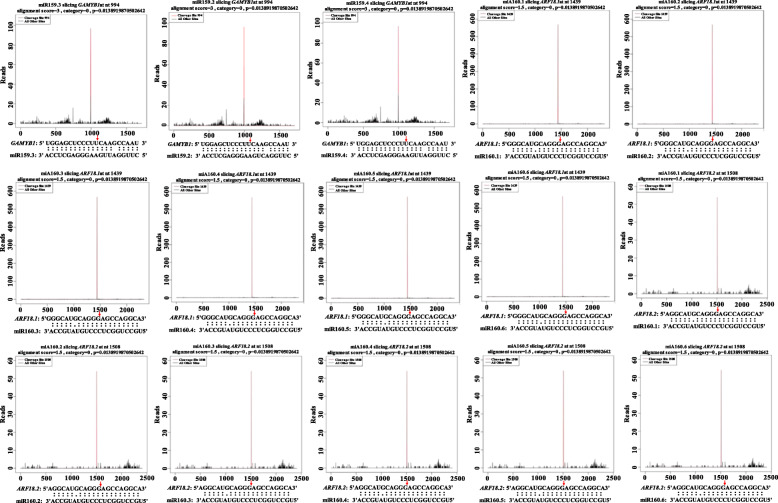
Validation of important miRNA-mRNA interaction pairs. The red lines and arrows represent the cleavage nucleotide positions on the target genes

**Fig. 6:**
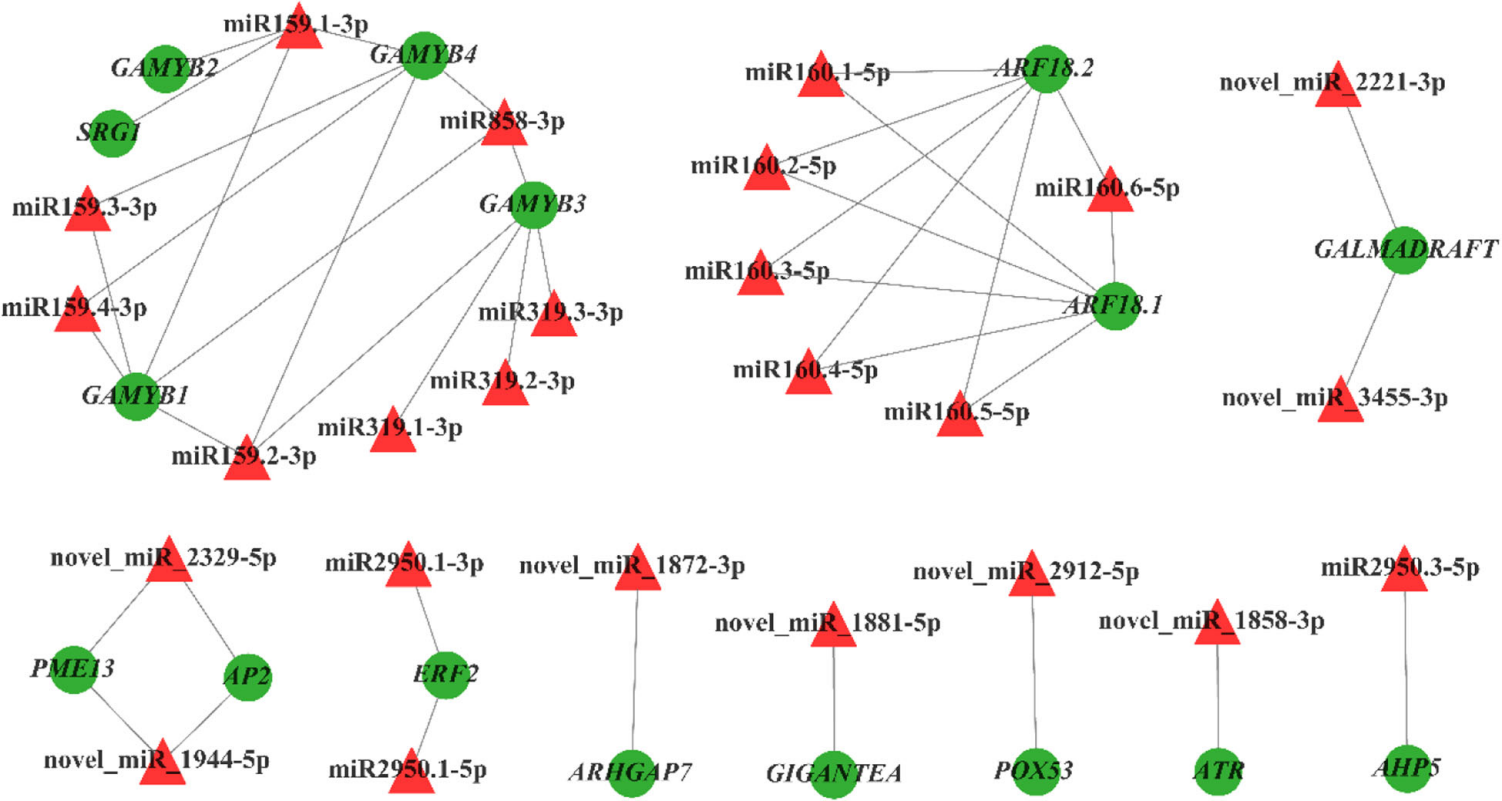
Network of relationships between miRNAs and target genes associated with strobilus development and sexual differentiation. Red triangles represent miRNAs and green circles represent target genes

A total of 2.283 million clean data were obtained by degradome sequencing from *G. biloba* strobili (Additional file 5: Table S3). Based on known miRNAs library, the prediction of miRNAs in the small RNA sequencing database, gene transcript sequence information files of corresponding species, and degradation site detection was performed by Cleaveland software [33]. The analysis revealed 155 degraded target genes and 156 target gene degradation sites. According to the integration analysis of degradome sequencing and the previous analysis results (Additional file 6: Table S4), fifteen miRNA-mRNA regulatory relationships were verified from the degradome sequencing, including three target genes and nine miRNAs (Fig. 5). miR159.2-3p, miR159.3-3p and miR159.4-3p jointly regulated *GAMYB1* involved in the GA metabolic pathway. miR160.1-5p, miR160.2-5p, miR160.3-5p, miR160.4-5p, miR160.5-5p and miR160.6-5p jointly regulated the *ARF18.1* and *ARF18.2* involved in the metabolic pathway of IAA (Fig. 6). There was only one miRNA degradation site on the three target genes, and the degradation site of *GAMYB1* was 994, the site of *ARF18.1* was 1439, and the site of *ARF18.2* was 1508. These results verified that multiple miRNAs could simultaneously regulate the same target gene.

### Validation of miRNA and its target gene by RT-qPCR

**Fig. 7:**
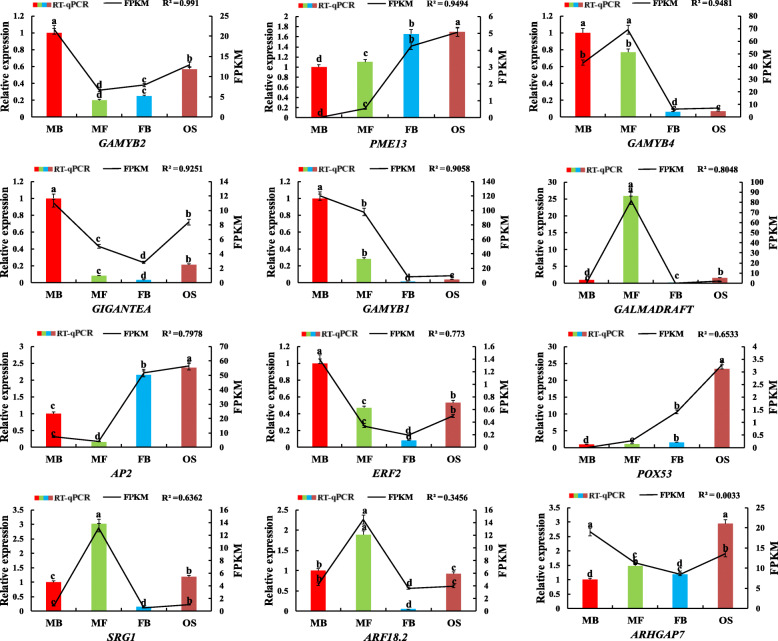
Expression levels of 12 target genes involved in the regulation of *G. biloba* sexual differentiation. The error bars represent the standard error of three biological replicates. Bar and line charts represent the RT-qPCR and FPKM values of the genes, respectively. The expression in MB of *G. biloba* was set as 1 and the different letters indicates values are significantly different at *P* < 0.05. The R^2^ value represents the correlation between the RT-qPCR and FPKM values

**Fig. 8:**
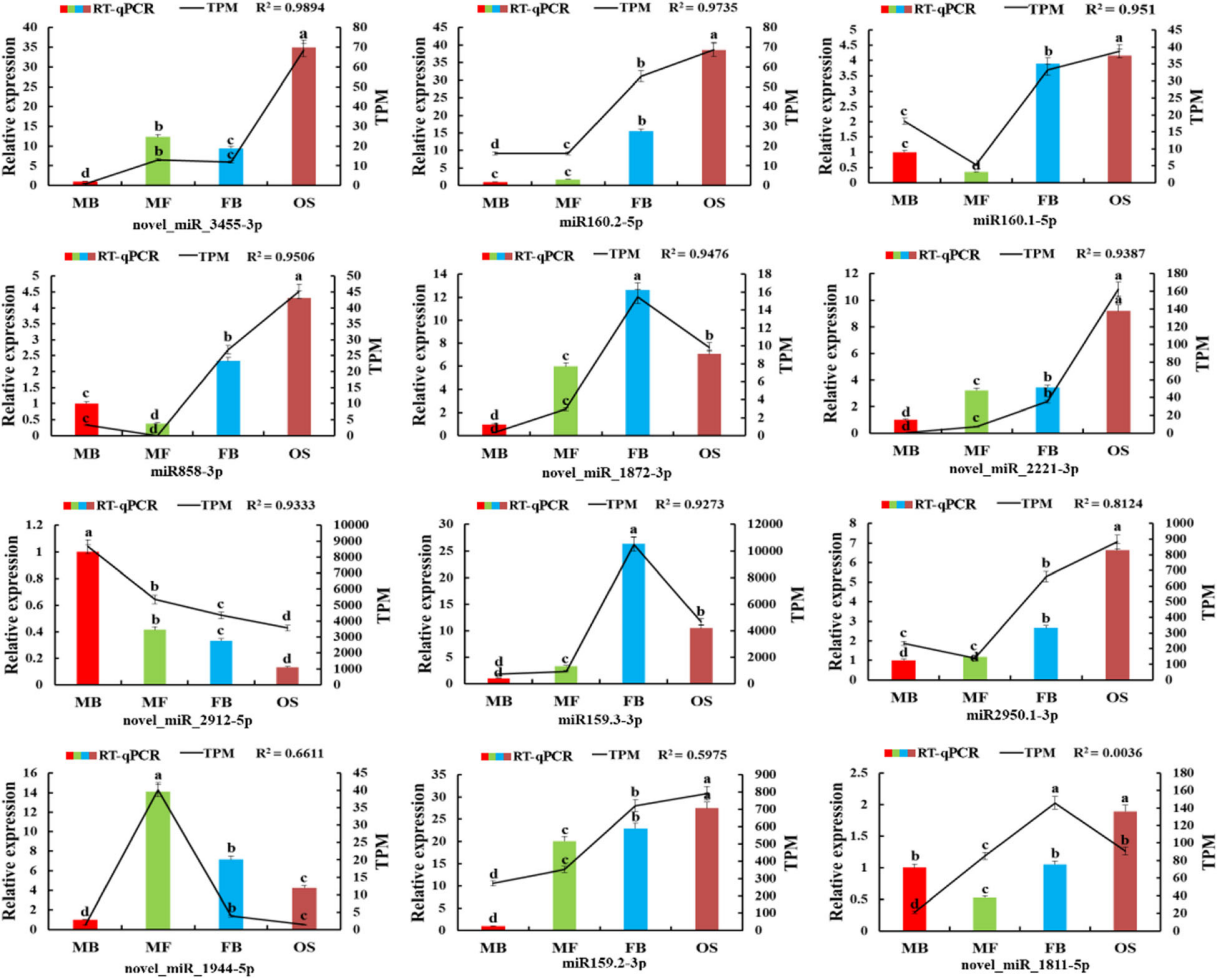
Expression levels of 12 miRNA involved in the regulation of *G. biloba* sex differentiation. The error bars represent the standard error of three biological replicates. Bar and line charts represent the RT-qPCR and TPM values of the genes, respectively. The expression in MB of *G. biloba* was set as 1 and the different lowercase letters indicates values are significantly different at *P* < 0.05. The R^2^ value represents the correlation between the RT-qPCR and TPM values

For verifying the identified expression patterns of miRNAs and corresponding target genes involved in the process of sexual differentiation of *G. biloba* strobili, 12 miRNAs and 12 mRNAs were randomly selected for RT-qPCR analysis. We compared the expression data of the four groups obtained by RNA-seq and RT-qPCR (Figs. 7 and 8). The correlation between RNA-Seq (FPKM/TPM) and qPCR (2^-ΔΔCt^) results for the genes and miRNAs was calculated using log2-fold variation measurements. The expression change trend of most genes and miRNAs observed from RT-qPCR analysis was similar to that of high-throughput sequencing. Among the 12 genes, the correlation value (R^2^) of 10 genes was between 0.6362 and 0.991 (Fig. 7). For miRNAs, R^2^ of 10 miRNAs was ranging from 0.661 to 0.9894 (Fig. 8). To sum up, these data revealed that there was good consistency between the high-throughput sequencing and RT-qPCR, indicating the reliability and accuracy of small RNA and mRNA transcriptome sequencing.

## Discussion

### Candidate miRNA-mRNA interactions participated in sex differentiation in *G. biloba*

**Fig. 9:**
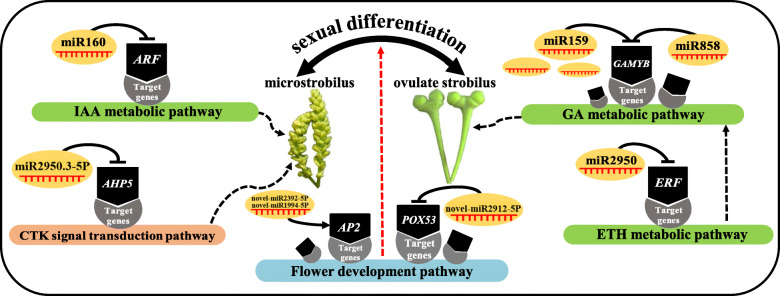
A model that miRNA regulating strobili sexual differentiation in *G. biloba.* Black dashed arrows indicate the promotion effect. T-shaped indicates inhibition. Red dashed arrow indicates that the corresponding miRNA has effect on strobili sex differentiation, but the promoting or inhibiting effect is unclear. The dashed black arrow indicates that ETH may act downstream of GA. The figure is freely available to use

Several works reported screening and characterization of a number of miRNAs from *G. biloba* using high-throughput sequencing, which potential participated in seed germi seed germination [34], ovules development [3], primary metabolisms in leaves [35], and terpene lactone biosynthesis [36]. Nevertheless, little is known about the miRNAs involved in sex differentiation of *G. biloba* strobili to date. Recently, Du et al. [37] used transcriptome data analysis to screen some genes related to sex differentiation in Ginkgo. However, single transcriptome sequencing cannot fully illuminate the gene regulatory network involved in plant sex differentiation. The combined analysis of multiple sequencing could present itself as a versatile tool to analyze the mechanisms of plant life process. The complex mechanism of Ascochyta blight resistance in chickpea were analyzed by means of multi-group sequencing combination [38]. In *Camellia*, miR172-*AP2* and miR156-*SPLs* regulatory pairs were identified as key regulatory nodes to promote the diversity of double flower forms by the multi-group analysis [39]. In this study, three high-throughput approaches, namely, transcriptomics, small RNA and degradome sequencings were used to elucidate the mechanisms of sexual differentiation in *G. biloba*. In addition, we identified miRNA-mRNA interaction pairs that may regulate the sex differentiation. Individual miRNA alone has no function and is easy to degrade. Only when miRNA combines with the Argonaute (AGO) protein to form the RNA-induced silencing complex (RISC) it can play an effective role, in which miRNA guides RISC to target-specific mRNA regions [40]. Plant miRNA can completely complement the coding regions of mRNAs, and inhibit the expression of genes by inducing the degradation of the complementing mRNAs [41]. A total of 51 miRNA-mRNA negative correlation regulatory relationships were identified in this research. The results of the present work indicated that miRNA might regulate sex differentiation by targeting transcription factor genes involved in the biosynthesis and signal transduction pathways of hormones, as well as flower development-related genes in *G. biloba* (Fig. 9).

### Sexual differentiation of *G. biloba* might be affected by miRNAs involved in ETH and GA biosynthesis pathways

ETH, one of plant hormones played important role in plant sexual determination, acts on the downstream of GA. ETH is a factor that promoted the development of female organs and inhibited the development of male organs [42]. Here, miR2950.1-3p and miR2950.1-5p targeted ethylene response transcription factor 2 (*EMF2*), which was down-regulated in FB of *G. biloba*, as opposed to the results of other plants [42]. The differences in regulatory function in sex differentiation might be due to the different species studied.

MYB is a large family of transcription factors in plants and involved in many life activities such as plant secondary metabolism [43], hormone responses [44], leaf organ formation [45], flower development [46]. At present, some MYB transcription factors have been identified as regulators of flower development [47]. In *A. thaliana*, several MYBs were reported to be involved in jasmonic acid-mediated stamen maturation. Transcriptional analysis indicated that MYB108 and MYB24 have overlapping functions and acted downstream of MYB21 in a transcriptional cascade that mediated stamen and pollen maturation [48, 49]. Our findings was consistent with the results of previous studies, which demonstrated that GA could promote differentiation of male flowers [8]. The present study found that miR159.1-3p, miR159.2-3p, miR159.3-3p, miR159.4-3p and miR858-3p were identified to target *GAMYB1* in MB, FB, MS and OS. The *GAMYB1* was down-regulated in FB and OS, indicating that the above-mentioned miRNAs target *GAMYB1* gene to make *GAMYB1* up-regulated in male strobili, thus promoting the maturation of *G. biloba* stamens. Moreover, it is well known that GA promotes the differentiation of the pistil. For example, maize, a monoecious plant, showed a phenotype in which the female flowers were transformed into male flowers or the flower primordium was biased toward pistil differentiation as a result of disruption of the GA signal transduction pathway or gene mutation during GA biosynthesis. These results also indicated endogenous GA promoted female flower differentiation in maize plants [42]. Likewise, our data also indicated that the *PME13* gene participated in the biosynthesis of GA and was up-regulated in OS. In addition, the content of GA_3_ in OS was significantly higher than that in MS, indicating that the expression level of this gene positively correlates with the GA_3_ content in strobili of *G. biloba*. Therefore, these results suggested that GA was likely to play a role in producing female strobili in *G. biloba*.

### Sexual differentiation might be affected by miRNAs functioned as regulator of IAA biosynthesis in *G. biloba*

IAA plays a key role in plant flower development such as flower primordia formation and flower organ differentiation [50]. In particular, most studies demonstrated that IAA promotes the expression of female flowers in plants [51]. For example, several *ARFs* and *Aux/IAAs* in IAA metabolic pathway have been identified to be essential for flower development in sugar apples [52]. In *A. thaliana*, *ARF3* integrates the functions of *AGAMOUS* and *APETALA2* in determining the flower meristem [53]. At the same time, two other members (*ARF6* and *ARF8*) of ARF family were verified to play an important role in controlling the growth and development of plant vegetative organs and flower organs [54]. Furthermore, studies have shown that the loss of *ARF18* activity causes the cell expansion in the silique wall, further promoting the development of siliques, increasing the photosynthetic products in siliques, and ultimately increasing the weight of the seeds [55]. In this study, six members of known miR160 family were identified to target *ARF18.1* and *ARF18.2* genes, which were differentially expressed only in MS _vs_ OS and were down-regulated in OS. And that, the expression level of miR160s is significantly negatively correlated with the expression level of the target gene identified in IAA biosynthesis among different organs (Table 2). Given that the IAA content in MS was higher than that in OS (Fig. 4), it can be speculated that miR160s participate in formation of male strobili through regulating IAA metabolism in *G. biloba*.

### miRNA regulating CTK signal transduction pathway affects sexual differentiation

Accumulated studies indicated that significant difference in CTK was usually detected between male and female flowers [56–58]. For example, treatment with exogenous CTK on *Mercurialis annua* L., a dioecious plant led the flower primordia to differentiate into pistil [56]. Similarly, treating exogenous CTK to the male flowers of spinach, hemp, grape and other plants caused changing of the male flowers into female flowers, indicating that CTK could promote the differentiation of flower primordia into female flowers [59]. One of the reason of effect CTK on plant sex differentiation could be regulatory function of sex-determining genes. For example, *SyGI*, the first sex-determining gene from kiwifruit, was found to inhibit gynoecial phenotype of through negatively regulating cytokinin signals [9]. In this study, miR2950.3-5p was identified to potentially regulate the signal transduction of CTK (Table 2). Moreover, miR2950.3-5p was differentially expressed only in MS _vs_ OS with up-regulation in OS and target *AHP5* gene was reciprocally expressed. Given that the contents of ZT in the male strobili in initial and final stage was higher than that in the female strobili (Fig. 4), our data implied that miR2950.3-5p affected the metabolism of CTK, and then may promote the development of male strobili and inhibit the formation of female strobili in *G. biloba*.

### miRNAs involved in regulating flower development pathway affect sexual differentiation of *G. biloba*

Plants have no obvious secondary sexual characteristics, and the difference between male and female sex is mainly manifested in floral organs [60]. *G. biloba* is a typical dioecious plant, so genes related to flower development must participate in the process of sexual differentiation. MADS-box genes are transcription factors present in a high number and have conservative characteristics. They participate in almost all kinds of pathways to regulate plant development especially for flower development [61]. Most of the genes in the ABCDE model related to flower development also belong to MADS-box gene family. AP2, one of MADS-box transcription factor, was consider to function in regulation of the flowering time, regulation of the development of seeds and maintenance of the ability of plant apical meristem [62, 63]. In the case of MS _vs_ OS, two novel miRNAs (novel_miR_1944-5p and novel_miR_2329-5p) simultaneously targeted the *AP2* gene, which was up-regulated in OS. At the same time, the novel_miR_1858-3p-*ATR* interaction pair was identified based on analysis of degradome sequencing. *ATR* was up-regulated expressed in OS of *G. biloba* and was annotated to participate in flower development pathway. In addition, two other novel miRNAs (novel_miR_1881-5p and novel_miR_2912-5p) were also found to be targeted genes related to flower development with up-regulated *GIGANTEA* and down-regulated *POX53* in FB, respectively (Table 2). Taken together, our results inferred that the interaction between these miRNAs and target genes played a regulatory role in sexual differentiation of *G. biloba*. It can regulate sexual differentiation by regulating not only genes related to flower development directly, but also transcription factors indirectly.

## Conclusion

This study is the first attempt to combine mRNA and miRNA expression data with degradome analysis to identify miRNAs involved in the regulation of sexual differentiation in *G. biloba*. Integration analysis of small RNA and mRNA were performed to identify 51 pairs of miRNA-mRNA interaction that may participate in regulating sexual differentiation in *G. biloba*. The finding was based on the screening of differentially expressed miRNA in combination with factors affecting sexual differentiation. The corresponding target mRNA also showed differential expression in male and female strobili of *G. biloba*. In total, 15 pairs among 51 miRNA-mRNA interaction pairs were confirmed by degradome sequencing. The comprehensive datasets of differential expression patterns of genes and miRNAs in male and female strobili presented a new insight to the sexual differential in *G. biloba*, and these sequencing data will also be a valuable resource for further study on *G. biloba*.

## Methods

### Plant material and RNA extraction

Thirty-two-year-old trees of *G. biloba* cv. ‘Jiafoshou’ is grown in the Ginkgo Garden of Yangtze University, China (30.35 °N, 112.14 °E). For the investigation of morphogenesis, the male and female strobili of *G. biloba* was observed and recorded with a digital camera from the March 8 to the April 12. The MB and FB harvested on March 13 as well as MS and OS harvested on April 5 were collected from *G. biloba* for extraction of total RNA to construct a sequencing library, and for RT-qPCR analysis and determinate the content of the hormone. A total nine independent Ginkgo trees were selected for the experiment, with 36 tree branches as the experimental block. Each biological replicate composed the samples selected from 12 independent branches from three ginkgo trees uniform in terms growth condition. A total of two biological repeats for the sequencing, and three biological repeats for the determination of the hormones content and the quantitative real-time PCR. The samples were collected, frozen in liquid nitrogen and stored in a refrigerator at − 80 °C for further analysis. The total RNA was extracted to construct a sequencing library and perform RT-qPCR analysis using a MiniBEST Universal RNA Extraction Kit (TakaRa, Dalian). The RNA samples were quantified, and the quality was assessed by a Nanodrop™ One spectrophotometer (ThermoFisher, America) and an Agilent 2100 Bioanalyzer (Agilent Technologies, America). RNA meeting the standard (OD260/280 ≥ 1.8, od260/230 ≥ 1.0. The RIN value of total RNA ≥8.0, 28S/18S ≥ 1.5, the baseline of the map is not lifted, and the 5S peak is normal.) is qualified sample.

### Scanning electron microscopy

The morphological of *G. biloba* cones were observed by SEM of the fresh MS and OS harvested on April 5. The ultrastructural analysis of MS and OS was performed according to the method described by Jin et al. [64] with following modification. The fresh samples were quickly fixed using 2.5% glutaraldehyde at 25 °C for 4 h and transferred to a refrigerator at 4 °C for 12 h. The fixed samples were taken out, and rinsed 2–3 times with 0.1 M phosphate buffer and then were dehydrated in a graded ethanol series (30, 50, 70, 80, 90, and 100%, 15 min each step). The dehydrated materials were transferred to a clean beaker, and immersed under isoamyl acetate twice for 15 min each time. After that, the samples were placed into a critical point drying device (high-pressure closed container) with injecting liquid carbon dioxide at a critical state of 31 °C and at 72.8 standard atmosphere pressures. The samples were coated with a layer of gold and were observed under 15 kV accelerated voltage using QUANAT-200 scanning electron microscope.

### Determination of phytohormone content

The extraction and content determination of IAA, GA_3_ and ZT according to the method described by Onanuga et al. [63] with following modification. The preparation sample was as below: 5 g of fresh sample was ground into powder 4 °C, and then added 50 ml of precooled 80% methanol solution. The mixture of sample powder and methanol solution was stirred for 12 h, ultrasonically crushed at 4 °C for 30 min, and then centrifuged at 12000 g/min for 10 min at 4 °C. Subsequently, the supernatant was extracted and concentrated to 10 mL using a rotary evaporator under reduced pressure at 35 °C; added equal volume petroleum ether for extraction for 3 times, and discard the organic phase. The aqueous extract were adjusted to pH 8.5 with 0.1 mol/L Na_2_HPO_4_ solution, and 0.2 g PVPP was added to shake well. After removing residue using a 0.45 μm filter membrane, the filtrate of aqueous extract was adjusted to pH 3.0 with 0.1 mol/L citric acid. Added equal volume of ethyl acetate for extraction for 3 times, discard the water phase, concentrate the remaining organic phase to about 2 mL using a rotary evaporator under reduced pressure at 35 °C, dissolved with methanol (chromatographic grade), and fixed the volume to 10 mL, then filtered with a 0.45 μm filter membrane to obtain the sample solution. High performance liquid chromatography (HPLC, Thermo Scientific™ UltiMate™ 3000, USA) with an Agilent C-18 chromatographic column (5 μm, 4.6 mm × 250 mm) was used for quantitative analysis of IAA, GA3 and ZT [65] with following modification. The mobile phase was methanol-1% acetic acid (40:60); flow rate: 0.6 mL/min; column temperature: 35 °C; injection volume: 2 μL; detection at 269 nm. Each sample was determined in six technical replicates.

### Small RNA library preparation and sequencing

In total, eight RNA samples (MB-1, MB-2, FB-1, FB-2, MS-1, MS-2, OS-1, OS-2) were subjected to small RNA sequencing. The library was constructed using the Small RNA Library Prep Kit as following steps. T4 RNA Ligase 1 and T4 RNA Ligase 2 (truncated) were used to ligate the 3′-SR and 5′-SR Adaptor. Then, reverse transcription synthetic first chain and the PCR amplification were performed. PAGE gel was used to electrophoresis fragment screening purposes, rubber cutting recycling as the pieces get small RNA libraries. The library quality was assessed on the Agilent Bioanalyzer 2100 system. The raw reads obtained by sequencing contained adapter sequence or low-quality reads. In order to ensure the accuracy of information analysis, a series of quality control checks were carried out on the raw reads using custom Perl script to obtain clean reads, including removing reads containing adapter, reads containing ploy-N and low-quality reads from raw data. The reads were trimmed and cleaned by removing the sequences smaller than 18 nt or longer than 30 nt. All the downstream analyses were based on clean data with high quality. The transcriptional level of small RNA was quantified according to Transcripts Per Kilobase of exon model per Million mapped reads (TPM) method [66]. The clean reads were compared with Silva database, GtRNAdb database, Rfam database and Repbase database, respectively, by using Bowtie software (v1.0.0, −v 0), and ncRNA such as ribosomal RNA (rRNA), transport RNA (tRNA), small nuclear RNA (snRNA), small nucleolar RNA (snoRNA) and repetitive sequences were filtered [67]. The remaining reads were used to detect known miRNA from miRbase (http://www.mirbase.org/), and predict novel miRNA by comparing with the reference Genomic seuqence of *G. biloba* [68]. The unique reads ranging from18–25 nt were collected and mapped to the reference genome by using the Bowtie software (v1.0.0, −v 0). All miRNA sequencing data are deposited in the NCBI SRA database (PRJNA590093).

### Transcriptome library construction and screening of DEGs

Eight RNA samples same with samples subjected to small RNA sequencing was used for the mRNA transcriptome sequencing. The RNA-seq libraries were constructed using NEBNext® Ultra™ RNA Library Prep kit (NEB, E7530L, USA) according to the kit instruction. The raw reads were obtained by sequencing of Illumina HiSeq4000 platform. High quality sequences (clean reads) were obtained by eliminating low-quality reads and removing contaminated adapter and removing reads with an N ratio greater than 5% [69]. The clean reads were then mapped to the reference genome sequence by using Hisat2 tools soft to obtain the Mapped reads, which were further analyzed and annotated based on the reference genome sequence of *G. biloba* [68]. DEseq software (http://www.bioconductor.org/packages/release/bioc/html/DESeq.html) was applied to analyze differential expression gene (DEG) between sample groups [70]. Quantification of gene expression levels were estimated by fragments per kilobase of transcript per million fragments mapped (FPKM). The resulting *P* values were adjusted using the Benjamini and Hochberg’s approach for controlling the false discovery rate. Genes with an adjusted *P*-value < 0.05 found by DESeq were assigned as differentially expressed, and uses a Fold Change ≥2 and FDR < 0.01 as screening criteria to obtain differential expression gene sets between two samples. All transcriptome sequencing data are available in the NCBI SRA database (PRJNA590044).

### Degradome library construction

Total RNA from the female and male strobili of *G. biloba* on March 13 and April 5 was mixed for the construction of the degradome library according to the kit instruction of NEBNext Ultra II RNA Library Prep Kit (NEB, E7775, USA). The degradome library was sequenced using an Illumina Hiseq 4000 platform [71]. Degradome reads were filtered using custom Perl script. Clean tags and cluster tags were obtained by removing adaptors and filtering low-quality raw tags. The cluster tags were compared to the reference genome to obtain the distribution of the tags on the genome [68]. Cluster tags and Rfam databases were compared to annotate non-coding RNA, and unannotated reads were used for degradation site analysis. The reads mapping to the sense strand of transcriptome were processed using the CleaveLand v4.4 pipeline to predict miRNA cleavage sites [33].

### Identification of miRNAs

The miRDeep2 software package (v2.0.5, −g − 1 -l 250 -b 0) was used to compare reads mapped to the reference genome with the known miRNA precursor sequences in the miRBase database to identify the expression of the known miRNAs [72]. Bowtie was used to map preprocessed readings to genomic sequences, allowing no more than two mismatches and 15 valid alignments. The sequence length was set to 250 to predict the secondary structure of RNA. At the same time, the possible precursors were obtained by comparing the position information of reads on the genome. Based on the distribution information on the genome of reads (miRNA production characteristics, mature, star, loop) and precursor structure energy information (randfold, v2.0, −s 99; RNAfold, v2.1.7, default). Bayesian model was used to score, and the plant-specific scoring system is added to miRDeep-P to realize the identification of novel miRNAs [73]. Finally, the miRNA targeting information was used to measure the effectiveness of miRNA.

### Screening of differentially expressed miRNAs

A normalization factor calculated by DESeq R (v1.18.0, default) was used to normalize the raw read counts of miRNA followed by identification of differentially expressed miRNA [74]. FDR and FC were used as the criteria for screening differentially expressed miRNA, |log2 (FC)| > = 1; FDR < =0.001.

### Prediction of miRNA target genes and annotation of differentially expressed target genes

According to combination information of the known miRNAs and novel miRNAs, gene sequence of corresponding species, and high complementarity between miRNAs and their target genes, the prediction of target genes was performed using TargetFinder software (v1.0.0, −v 0) [75]. Using BLAST software (v2.2.26, −b 100 -v 100 -e 1e-5 -m 7 -a 2), the differentially expressed target genes were compared with the National Center for Biotechnology Information non-redundant (NCBI nr) [76], Swiss-Prot [77], Gene ontology (GO) [78], Kyoto Encyclopedia of Genes and Genomes (KEGG) [79] and Cluster of Orthologous Groups of proteins (COG) [80] databases for similarity (E-value 1e-05) to obtain annotation information.

### qRT-PCR validation of mRNA and miRNA

Forward specific primers were designed based on the sequence of mature miRNAs (Additional file 7: Table S5), and the reverse primers used the universal primers provided by the Mir-X miRNA First-Strand Synthesis Kit. The upstream and downstream primers (Tm: 60 °C) of the external gene U6 contained in the Mir-X miRNA First-Strand Synthesis Kit were used as the reference gene primers. The RT-qPCR reactions were performed using a TB GreenTM Advantage® kit (Takara, Dalian, China) in LineGene 9600 Plus (FQD-96A, China).

Specific primers of mRNA (sequences in Additional file 8: Table S6) were designed by software Primer 5.0 according to the sequences of the target genes. *G. biloba 18S* gene was used as the reference gene [81, 82]. The RT-qPCR reaction was executed according to the instruction manual of the AceQ® qPCR SYBR® Green Master Mix (Takara, Dalian). Experiments were performed with three independent biological triplicates and each triplicate consisted of three technical replicates. The expression of miRNAs and target gene mRNAs in flowers of *G. biloba* was calculated by the 2^-ΔΔCt^ method [83]. The standard error between biological replicates was analyzed by SPSS 22.0 (SPSS Inc., Chicago, IL, USA).

## Supplementary information

**Additional file 1: Table S1** Identification of known and novel miRNAs.

**Additional file 2: Figure S1** Length distribution and characterization of small RNA by deep sequencing. (A) Length distribution of small RNA sequences. (B) The first nucleotide bias of miRNA with different predicted lengths. (C) The relative nucleotide bias at each position of the miRNAs.

**Additional file 3: Table S2** Differentially expressed miRNAs between female and male strobili of two stages in *G. biloba*.

**Additional file 4: Figure S2** Functional annotation of target genes of DE miRNAs. (A) Histogram of GO classification. (B) COG functional classification. (C) List of pathway enrichment analysis. (D) Scatter diagram of KEGG pathway enrichment.

**Additional file 5: Table S3** Statistics of Ginkgo degradome sequencing data.

**Additional file 6: Table S4** Integrated information of combined analysis of small RNA, transcriptome and degradome sequencing.

**Additional file 7: Table S5** Primers for miRNA detection by RT-qPCR.

**Additional file 8: Table S6** Primers for target genes detection by RT-qPCR.

## Data Availability

The datasets generated and/or analysed during the current study are available in the NCBI repository, [NCBI SRA database (PRJNA590044) and NCBI SRA database (PRJNA590093)]. All data generated or analysed during this study are included in this published article Additional file. The datasets used and/or analyzed during the current study are available from the authors on reasonable request (Feng Xu, xufeng198@126.com; Xiao-Meng Liu, LiuXM925@163.com).

## Abbreviations

DEGs: Differentially expressed genes
MB: Male buds
FB: Female buds
MS: Microstrobilus
OS: Ovulate strobilus
SLY1: Y-gene 1
SyGI: Shy Girl
FrBy: Friendly boy
IAA: Indoleacetic acid
ETH: Auxin, ethylene
GA: Gibberellin
CTK: Cytokinin
miRNA: microRNA
SEM: Scanning electron microscope
ZT: Zeatin
ACS2: 1-aminocyclopropane-1-carboxylate synthase
CmWIP1: C2H2 zinc finger transcription factors named WIP proteins
bZIP48: Basic region-leucine zipper
DCL1: DICER-LIKE1
GbCO: CONSTANS
GbFT: FLOWERING LOCUS T
RT-qPCR: Quantitative real-time RCR
FPKM: Fragments per kilobase of transcript per million fragments mapped
SPL: SQUMOSA PROMOTER BINDINGPROTEIN LIKE
AGO: Argonaute
RISC: RNA-induced silencing complex
EMF2: Ethylene response transcription factor 2
AP2: APETALA2
PVPP: Polyvinylpolypyrrolidone
HPLC: High performance liquid chromatography
TPM: Transcripts Per Kilobase of exon model per Million mapped reads
rRNA: Ribosomal RNA
tRNA: Transport RNA
snRNA: Small nuclear RNA
snoRNA: Small nucleolar RNA
NCBI: National Center for Biotechnology Information
FDR: False Discovery Rate
FC: Fold Change
NCBI nr: National Center for Biotechnology Information non-redundant
GO: Gene ontology
KEGG: Kyoto Encyclopedia of Genes and Genomes
COG: Cluster of Orthologous Groups of proteins
