# Supplementary material for: Screening and identification of miRNAs related to sexual differentiation of strobili in Ginkgo biloba by integration analysis of small RNA, RNA, and degradome sequencing
Source: BMC Plant Biol. 2020 Aug 25;20:387. doi: 10.1186/s12870-020-02598-8 (PMC7446137; doi:10.1186/s12870-020-02598-8)
Supplement: Supplementary file 5 — Additional file 5: Table S3 Statistics of Ginkgo degradome sequencing data. [file 12870_2020_2598_MOESM5_ESM.docx]

**Table S3** Statistics of Ginkgo degradome sequencing data

| **Samples** | **BMK ID** | **Species** | **Clean Data** | **Tags numbers** | **Q30(%)** |
| --- | --- | --- | --- | --- | --- |
| MB-1/-2 | D01 | *Ginkgo biloba* | 22,830,834 | 7,226,416 | 96.36 |
| MS-1/-2 |  |  |  |  |  |
| FB-1/-2 |  |  |  |  |  |
| OS-1/-2 |  |  |  |  |  |
