# Supplementary material for: Screening and identification of miRNAs related to sexual differentiation of strobili in Ginkgo biloba by integration analysis of small RNA, RNA, and degradome sequencing
Source: BMC Plant Biol. 2020 Aug 25;20:387. doi: 10.1186/s12870-020-02598-8 (PMC7446137; doi:10.1186/s12870-020-02598-8)
Supplement: Supplementary file 7 — Additional file 7: Table S5 Primers for miRNA detection by RT-qPCR. [file 12870_2020_2598_MOESM7_ESM.docx]

**Table S5** Primers for miRNA detection by RT-qPCR

| **mi RNA ID** | **Primer** | **Primer sequences (5' to 3')** |
| --- | --- | --- |
| miR2950.1-3p | Forward | CGTTCCATCTCTTGCACCCTCC |
| miR160.1-5p | Forward | TGCCTGGCTCCCTGTATGC |
| miR159.2-3p | Forward | CTTGGACTGAAGGGAGCTCCA |
| miR159.3-3p | Forward | GCTTGGATTGAAGGGAGCTCCA |
| miR858-3p | Forward | GTTCGTTGTCTGTTCGGCCTG |
| novel_miR_1872-3p | Forward | CTTGCTGCGTCTGTACATGGC |
| novel_miR_2221-3p | Forward | ATTGGGGGCGTTGGGGGAGAA |
| novel_miR_3455-3p | Forward | GGGGCGTTGGGGGAGACTT |
| novel_miR_1944-5p | Forward | GCCTTCCCATGTTGCCACC |
| novel_miR_1881-5p | Forward | CCGCCAACTGCATTTCATGATGCT |
| novel_miR_2912-5p | Forward | GCTGAAAAGGAAGGCAAGAGCAG |
| miR160.2-5p | Forward | CGCTTCCATCTCTTGCACACC |
