# Supplementary material for: Screening and identification of miRNAs related to sexual differentiation of strobili in Ginkgo biloba by integration analysis of small RNA, RNA, and degradome sequencing
Source: BMC Plant Biol. 2020 Aug 25;20:387. doi: 10.1186/s12870-020-02598-8 (PMC7446137; doi:10.1186/s12870-020-02598-8)
Supplement: Supplementary file 8 — Additional file 8: Table S6 Primers for target genes detection by RT-qPCR. [file 12870_2020_2598_MOESM8_ESM.docx]

| **Metabolic pathways** | **Gene name** | **Gene ID** | **Primer** | **Primer sequences (5' to 3')** |
| --- | --- | --- | --- | --- |
| ETH biosynthetic pathway | *ERF2* | gene.Gb_34844 | Forward | TCATCAGACGGGAATGGAGACAC |
|  |  |  | Reverse | CGGGCTAACAGTCGCTTCTCTAA |
| IAA biosynthetic pathway | *ARF18.2* | gene.Gb_39786 | Forward | AGGATTACAAGCAGGGATTCAG |
|  |  |  | Reverse | AGGATTACAAGCAGGGATTCAG |
| GA biosynthetic pathway | *GAMYB1* | gene.Gb_11536 | Forward | GGCAACTTCTCTGCTTCTACACC |
|  |  |  | Reverse | CAGCAGGAGGAGACAGTGTAAATG |
|  | *GAMYB4* | gene.Gb_23921 | Forward | ACGCTAAACTTGGCAACAAATGG |
|  |  |  | Reverse | CATCAGGCGGATACAGAGAAAGC |
|  | *GAMYB2* | gene.Gb_10456 | Forward | GAGAATGAAACTCCTGGCGGTG |
|  |  |  | Reverse | GAGAATGAAACTCCTGGCGGTG |
|  | *ARHGAP7* | gene.Gb_24678 | Forward | GAGACACCGACCTGATTCTATG |
|  |  |  | Reverse | TATGGATCCCAGGCTCATTTG |
|  | *SRG1* | gene.Gb_17508 | Forward | TGCTGTATGGGCAGGTTATG |
|  |  |  | Reverse | GTGCTTCCGTCATACTGTAGTT |
|  | *PME13* | gene.Gb_07887 | Forward | CAGACCACAGTCCATTCTTCTC |
|  |  |  | Reverse | CATCATCTCTAGCCGCACATAA |
|  | *GALMADRAFT* | gene.Gb_23928 | Forward | GACGCTACCAATCCCACTAAA |
|  |  |  | Reverse | CGTTCTCTGGGTAGTCCAAATAG |
| Flower development pathway | *POX53* | gene.Gb_31013 | Forward | TGCGCTGATATCCTGACTATTG |
|  |  |  | Reverse | ATTTGCTGTCCTGCTATCTCTC |
|  | *GIGANTEA* | gene.Gb_17618 | Forward | GATCCCTCCTTTCAGCTCTTTC |
|  |  |  | Reverse | GGTCGGTTGTAGTGCGTTAATA |
|  | *AP2* | gene.Gb_30123 | Forward | CGGCTCATCTGCTCCAATTTA |
|  |  |  | Reverse | CTACCCGAGCTTTCTTCCAATC |
| Reference gene | *18S* | GenBank accession no. D16448 | Forward | ATAACAATACTGGGCTCATCG |
|  |  |  | Reverse | TTCGCAGTGGTTCGTCTTTC |

**Table S6** Primers for target genes detection by RT-qPCR
