# Supplementary material for: Screening and identification of miRNAs related to sexual differentiation of strobili in Ginkgo biloba by integration analysis of small RNA, RNA, and degradome sequencing
Source: BMC Plant Biol. 2020 Aug 25;20:387. doi: 10.1186/s12870-020-02598-8 (PMC7446137; doi:10.1186/s12870-020-02598-8)
Supplement: Supplementary file 7 [file 12870_2020_2598_MOESM2_ESM.docx]

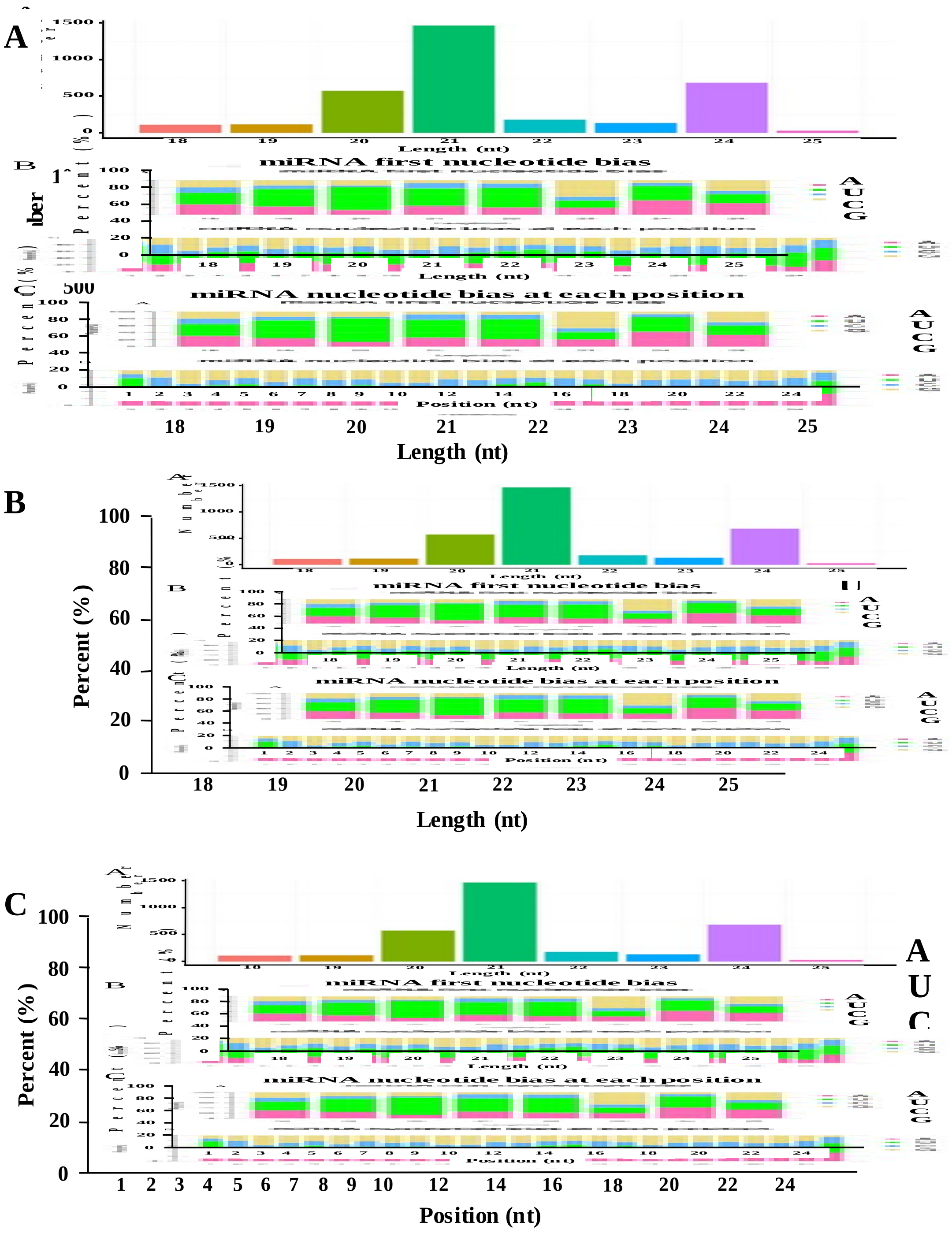
A
B
C
miRNA first nucleotide bias
miRNA nucleotide bias at each position
Length (nt)
Position (nt)
Percent (%)
Number
A
U
C
G
