# Supplementary material for: Screening and identification of miRNAs related to sexual differentiation of strobili in Ginkgo biloba by integration analysis of small RNA, RNA, and degradome sequencing
Source: BMC Plant Biol. 2020 Aug 25;20:387. doi: 10.1186/s12870-020-02598-8 (PMC7446137; doi:10.1186/s12870-020-02598-8)
Supplement: Supplementary file 8 [file 12870_2020_2598_MOESM4_ESM.docx]

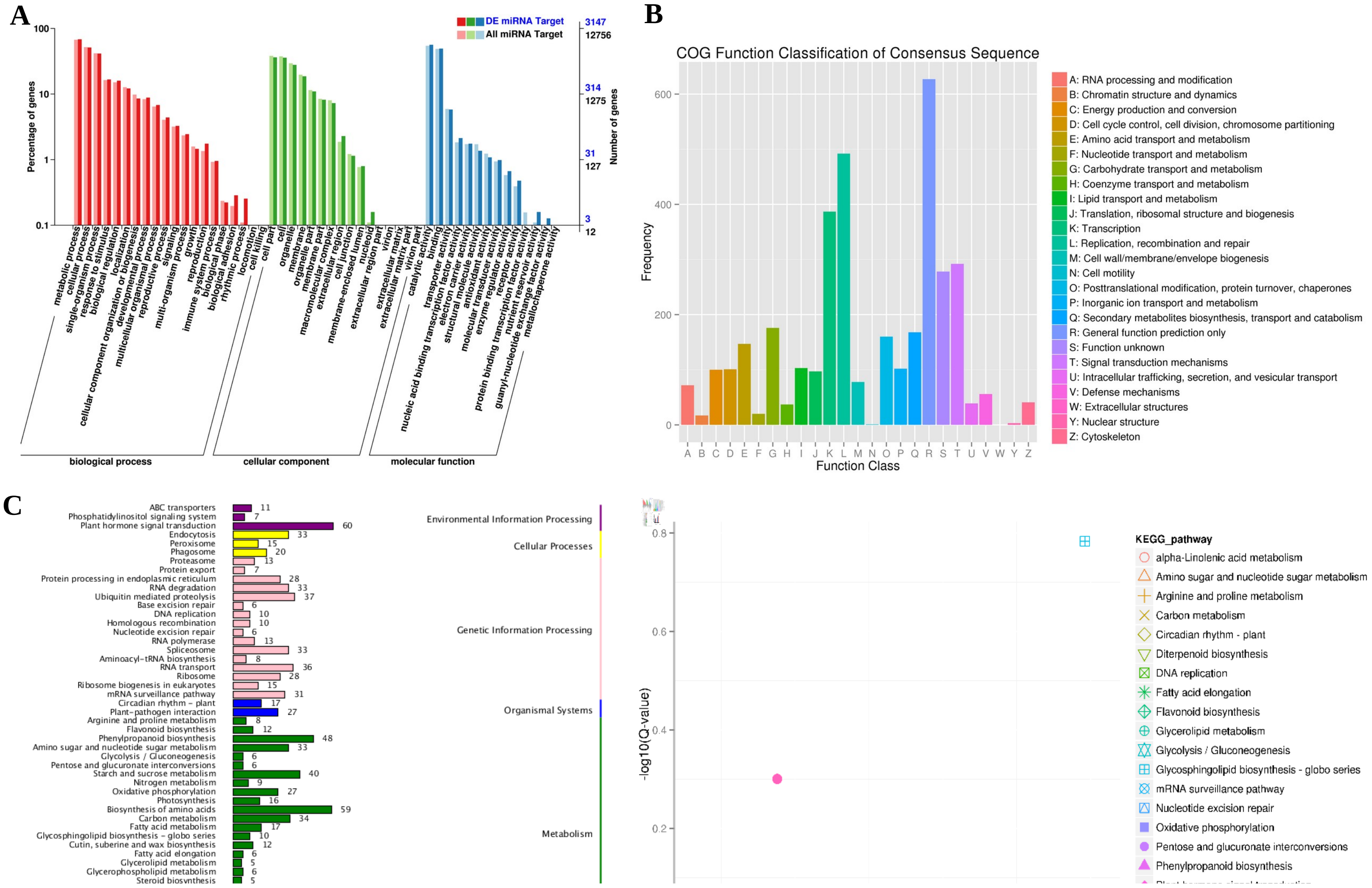
A
DE miRNA Target
All miRNA Target
Percentage of genes
Number of genes
100
10
1
0.1
3147
12756
314
1275
31
127
3
12
metabolic process
cellular process
single-organism process
response to stimulus
biological regulation
localization
cellular component organization or biogenesis
developmental process
multicellular organismal process
reproductive process
signaling
multi-organism process
growth
reproduction
immune system process
biological phase
biological adhesion
rhythmic process
locomotion
cell killing
cell part
cell
organelle
membrane
organelle part
membrane part
macromolecular complex
extracellular region
cell junction
membrane-enclosed lumen
nucleoid
extracellular region part
virion
extracellular matrix
extracellular matrix part
virion part
catalytic activity
binding
transporter activity
nucleic acid binding transcription factor activity
electron carrier activity
structural molecule activity
antioxidant activity
molecular transducer activity
enzyme regulator activity
receptor activity
protein binding transcription factor activity
nutrient reservoir activity
guanyl-nucleotide exchange factor activity
metallochaperone activity
biological process
cellular component
molecular function
B
COG Function Classification of Consensus Sequence
Frequency
600
400
200
0
A B C D E F G H I J K L M N O P Q R S T U V W Y Z
Function Class
A: RNA processing and modification
B: Chromatin structure and dynamics
C: Energy production and conversion
D: Cell cycle control, cell division, chromosome partitioning
E: Amino acid transport and metabolism
F: Nucleotide transport and metabolism
G: Carbohydrate transport and metabolism
H: Coenzyme transport and metabolism
I: Lipid transport and metabolism
J: Translation, ribosomal structure and biogenesis
K: Transcription
L: Replication, recombination and repair
M: Cell wall/membrane/envelope biogenesis
N: Cell motility
O: Posttranslational modification, protein turnover, chaperones
P: Inorganic ion transport and metabolism
Q: Secondary metabolites biosynthesis, transport and catabolism
R: General function prediction only
S: Function unknown
T: Signal transduction mechanisms
U: Intracellular trafficking, secretion, and vesicular transport
V: Defense mechanisms
W: Extracellular structures
Y: Nuclear structure
Z: Cytoskeleton
C
ABC transporters 11
Phosphatidylinositol signaling system 7
Plant hormone signal transduction 60
Endocytosis 33
Peroxisome 15
Phagosome 20
Proteasome 13
Protein export 7
Protein processing in endoplasmic reticulum 28
RNA degradation 33
Ubiquitin mediated proteolysis 37
Base excision repair 6
DNA replication 10
Homologous recombination 10
Nucleotide excision repair 6
RNA polymerase 13
Spliceosome 33
Aminoacyl-tRNA biosynthesis 8
RNA transport 36
Ribosome 28
Ribosome biogenesis in eukaryotes 15
mRNA surveillance pathway 31
Circadian rhythm - plant 17
Plant-pathogen interaction 27
Arginine and proline metabolism 8
Flavonoid biosynthesis 12
Phenylpropanoid biosynthesis 48
Amino sugar and nucleotide sugar metabolism 33
Glycolysis / Gluconeogenesis 6
Pentose and glucuronate interconversions 6
Starch and sucrose metabolism 40
Nitrogen metabolism 9
Oxidative phosphorylation 27
Photosynthesis 16
Biosynthesis of amino acids 59
Carbon metabolism 34
Fatty acid metabolism 17
Glycosphingolipid biosynthesis - globo series 10
Cutin, suberine and wax biosynthesis 12
Fatty acid elongation 6
Glycerolipid metabolism 5
Glycerophospholipid metabolism 6
Steroid biosynthesis 5
Environmental Information Processing
Cellular Processes
Genetic Information Processing
Organismal Systems
Metabolism
-log10(Q-value)
0.8
0.6
0.4
0.2
KEGG_pathway
alpha-Linolenic acid metabolism
Amino sugar and nucleotide sugar metabolism
Arginine and proline metabolism
Carbon metabolism
Circadian rhythm - plant
Diterpenoid biosynthesis
DNA replication
Fatty acid elongation
Flavonoid biosynthesis
Glycerolipid metabolism
Glycolysis / Gluconeogenesis
Glycosphingolipid biosynthesis - globo series
mRNA surveillance pathway
Nucleotide excision repair
Oxidative phosphorylation
Pentose and glucuronate interconversions
Phenylpropanoid biosynthesis
